# Primary tumor volume delineation in head and neck cancer: missing the tip of the iceberg?

**DOI:** 10.1186/s13014-017-0838-4

**Published:** 2017-06-20

**Authors:** Olgun Elicin, Dario Terribilini, Mohamed Shelan, Werner Volken, Etienne Mathier, Alan Dal Pra, Daniel M. Aebersold, Michael K. Fix, Peter Manser

**Affiliations:** Department of Radiation Oncology and Division of Medical Radiation Physics, Inselspital, Bern University Hospital, and University of Bern, Bern, Switzerland

**Keywords:** Head and neck cancer, Radiotherapy, Target volume, Contouring

## Abstract

**Background:**

The aim was to evaluate the geometric and corresponding dosimetric differences between two delineation strategies for head and neck tumors neighboring air cavities.

**Methods:**

Primary gross and clinical tumor volumes (GTV and CTV) of 14 patients with oropharynx or larynx tumors were contoured using a soft tissue window (S). In a second strategy, the same volumes were contoured with an extension to include the parts which became visible on lung window (L). For the calculation of Hausdorff-distances (HD) between contoured volumes of the two strategies, triangular meshes were exported. Two radiotherapy plans with identical goals and optimization parameters were generated for each case. Plan_S were optimized on CTV_S, and Plan_L on CTV_L. The dose coverages of CTV_L and CTV_Δ (CTV_L minus CTV_S) were evaluated in Plan_S. OAR doses were compared among Plan_S and Plan_L.

**Results:**

Median three-dimensional HD for GTVs and CTVs were 5.7 (±2.6) and 9.3 (±2.8) mm, respectively. The median volume differences between structures contoured using L and S windows were 9% (±5%) and 9% (±4%) for GTV and CTV, respectively. In 13 out of 14 cases, Plan_S met the plan acceptance criteria for CTV_L. In 8 cases CTV_Δ was covered insufficiently in Plan_S. Mean and median differences in OAR dose-volume histogram parameters between Plan_S and Plan_L were within 3%.

**Conclusion:**

For the current practice in radiotherapy planning for head and neck cancer, the delineation of L-based volumes seems unnecessary. However, in special settings, where smaller or no PTV margins are used, this approach may play an important role for local control.

**Electronic supplementary material:**

The online version of this article (doi:10.1186/s13014-017-0838-4) contains supplementary material, which is available to authorized users.

## Introduction

Currently, there is a well-established international consensus among radiation oncologists for the delineation of the organs at risk (OARs) and the elective lymphatic levels for the radiotherapy planning of squamous cell head and neck cancer (SCCHN) [1, 2]. Despite the heterogeneity based on the equipment and institutional protocols, image guided treatment technologies are allowing the clinicians to reduce the margins used to generate the planning treatment volumes (PTVs) around the clinical target volumes (CTVs). It is difficult to forecast, if and when the PTV can be abolished in the future (i.e. CTV = PTV). Before reaching this goal, there is another uncertainty which needs to be addressed: the uncertainty and heterogeneity in the delineation strategy of the high-risk CTV around the primary tumor [3].

There are different strategies to define this high-risk CTV, most of them based on tradition, data inferred from pathology results and patterns of failure. One method is based on pre-defined anatomical subsites [4], whereas the other approach uses an isotropic expansion around the gross tumor volume (GTV), which is later modified by anatomical barriers (e.g. air, non-infiltrated bone, fascia) [5]. Some radiation oncologists use a combination of both strategies [6]. It is worth to note, that the expansion margin also varies among radiation oncologists and treatment protocols. Kim et al. recently defined the CTV as the “third front” of radiotherapy as the field least investigated and methodologically most difficult to investigate among the target volumes [7].

For the visualization and delineation of structures situated in the lung parenchyma, lung window (L) is recommended and commonly used. It contrasts the soft-tissue structures against neighboring and surrounding air with higher fidelity over soft tissue window (S) [8]. The majority of SCCHN tumors arise from the mucosa and thus there is an air interface with the tumor. However, to our knowledge there is no literature recommending or investigating the use of the L for the delineation of target volumes for SCCHN.

This work aims to investigate the geometric and dosimetric impact of two different contouring strategies based on S and L in a hypothetical ‘ideal future scenario’, where PTV would be tighter around, or preferably equal to the CTV, if the systematic and random errors can be minimized or eliminated somehow [9].

## Methods

**Fig. 1:**
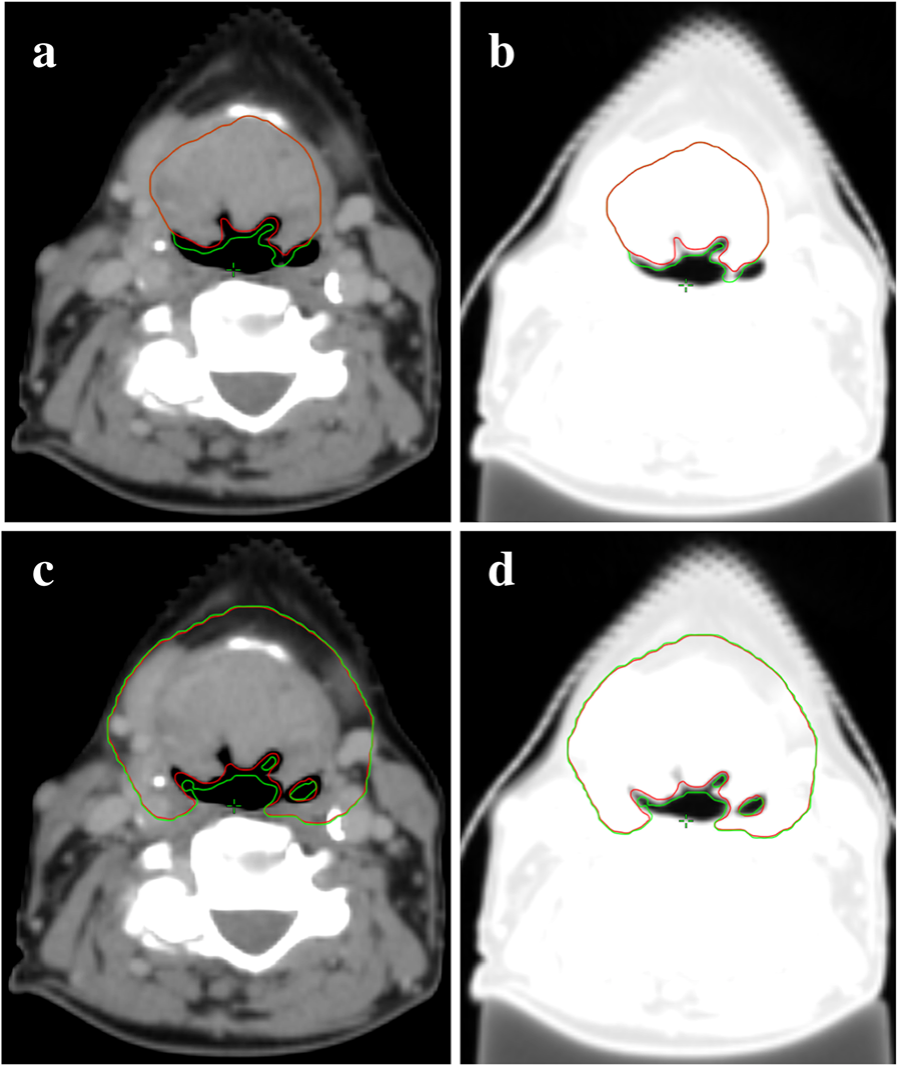
Differences in target volume delineation in soft tissue and lung windows. GTV and CTV contoured on lung window (*green* structures) are slightly larger than their counterparts contoured in soft tissue window (*red* structures). **a**: Both GTVs on soft tissue window; **b**: Both GTVs on lung window; **c**: Both CTVs on soft tissue window; **d**: Both CTVs on lung window

Planning-CT data sets of 14 previously treated SCCHN cases with primary tumors emerging from oropharynx or larynx were used for the study. A summary of patient and disease characteristics are provided in Table 1. Primary tumor GTVs were delineated using S (GTV_S). In all GTVs an extension towards the air was revealed by switching to L. GTVs were expanded to cover these additional parts which created GTV_L. Using an isotropic 8 mm margin, CTV_L was generated around GTV_L. Air, bone and anatomical boundaries were cropped out to finalize CTV_L. Based on GTV_S, the same process was repeated to generate CTV_S using S (Fig. 1). S corresponded to the default acquisition parameters used for head and neck imaging in our planning CT, and L was the default presets (Hounsfield Unit range: -1000 – 0) in the Eclipse treatment planning system (version 13.6, Varian Medical Systems, Palo Alto, CA).

**Table 1:** patient and disease characteristics

Characteristic	Value
Mean age (range)	63 (50–79)
Female/male	6/8
Tumor subsite	
Oropharynx	9
Supraglottic larynx	5
cT Stage^a^	
T2	6
T3	2
T4a	6
cN Stage^a^	
N0	3
N1	2
N2(a/b/c)	9 (0/2/7)

^a^UICC 7th edition. All patients were cM0

Triangular meshes (TMs) for all volumes were exported using the Eclipse Research Scripting API 13.6. An analysis-tool written in Python 3.5 was used to analyze the TMs and to calculate surfaces, volumes and Hausdorff-distances (HDs) between the two different contour sets. The Python scripts are available upon request to the corresponding author.

Two volumetric modulated arc therapy plans were generated for each case. Plan_S were optimized on CTV_S, and Plan_L on CTV_L. All plans were prescribed to 70 Gy normalized on D95%. The plan objectives (Table 2) and optimization criteria (Additional file 1) were identical to our institutional standards except for the use of CTV instead of PTV. The same OARs and corresponding dose constraints were used for the optimization of all plans (i.e. geometrical differences only existing between the L- and S-based target volumes).

**Table 2:** target volume objectives for dose coverage

Objective^b^	Per Protocol	Variation Acceptable	Deviation Unacceptable
D_95%_ of PTV70	70 Gy (100%)	≤+/-2%	>+/-2%
D_5%_ of PTV70	≤74.9 Gy (107%)	≤77 Gy (110%)	>77 Gy (110%)
D_98%_ of PTV70	≥66.5 Gy (95%)	≥63 Gy (90%)^a^	<63 Gy (90%)^a^
Maximum dose (hot spot ≤0.03 cm^3^) outside the PTV70	≤74.9 Gy (107%)	≤77 Gy (110%)	>77 Gy (110%)

^a^It is recognized that portions of the PTV close to the skin may receive significantly less than 90% of the prescribed dose. This is acceptable as long as cold spots within PTV do not exist at a depth deeper than 5 mm beneath the skin

^b^CTV_S and CTV_L used instead of PTV70 for the purpose of this study

To investigate any possible under-dose of the air-tumor surface, the dose coverage of CTV_L was assessed in Plan_S. Additionally, the dose coverage of CTV_Δ (CTV_L minus CTV_S) was determined. In order to quantify the dosimetric differences, OAR doses were compared among Plan_S and Plan_L.

In this study, the geometric and dosimetric differences between two target volume delineation strategies are evaluated. Thus, the statistical methodology was purely descriptive, because the differences in target volumes and dose coverage are unidirectional, where any “statistically significant” difference can be demonstrated anyway.

## Results

Volumes of the GTVs and CTVs resulted by applying the two contouring approaches are provided in Table 3. Median three-dimensional HD for GTVs and CTVs were 5.7 (±2.6) and 9.3 (±2.8) mm, respectively. Median two-dimensional HD for the same structures in axial plane were 4.7 (±2.8) and 5.2 (±3.8) mm, respectively. The median volume differences between L and S were 9% (±5%) and 9% (±4%) for GTV and CTV, respectively.

**Table 3:** gross and clinical tumor volumes

Structure	Median Volume in cm^3^ (*range*)
GTV_S	22.5 (4.3–49.3)
GTV_L	25.4 (5.6–54.9)
CTV_S	89.7 (30.6–166.2)
CTV_L	98.4 (34.1–176.5)

All planning objectives were successfully met for CTV_S in all Plan_S, and for CTV_L in all Plan_L. In 13 out of 14 cases, Plan_S met the plan acceptance criteria for CTV_L. However, in 8 cases CTV_Δ was covered with an inadequate dose (D98% as provided in Table 2) in Plan_S. Dose coverage of each CTV_L and CTV_Δ on Plan_S is provided in Table 4. Median differences in OAR doses between Plan_S and Plan_L were up to 2.4% with a minimum-maximum range of 0-17% (Additional file 1).

**Table 4:** dose coverages (%) of CTV_L and CTV_Δ on Plan_S

Case	CTV_L D_2_	CTV_L D_95%_	CTV_L D_98%_	CTV_Δ D_98%_
1	104.7	99.6	97.9	89.8^a^
2	104.3	99.8	98.2	96.7
3	104.5	99.7	97.9	95.6
4	104	99.6	97	94.1^a^
5	104.5	99.8	98.4	96.7
6	103.9	99.6	98.5	98.1
7	104.8	97.5^a^	94.8^a^	85.1^a^
8	104.3	99.5	97.9	94.1^a^
9	105	99.2	97.3	97.5
10	104.4	99.7	98.2	96.5
11	105.5	99.2	97	85.6^a^
12	105.7	99.4	97.3	67.1^a^
13	105.2	98.7	96.7	91.9^a^
14	105.3	99	97.1	92.5^a^

^a^considered as under-coverage (please be referred to the Additional file 1)

## Discussion

In this in silico analysis, a median increase of 9% in target volumes and a median three-dimensional HD close to 1 cm was observed. In over half of the cases, the CTV_Δ was covered with an inadequate dose. However, it is unknown whether this would reflect in a reduced tumor control probability (TCP) in reality. When looked at the dose coverage of the whole CTV_L and not just at the difference volume CTV_Δ, only one case (number 7 in Table 4) would receive an inadequate dose through the Plan_S.

It is worth to note, that the dosimetric results of this study, especially doses received by the OARs, do not reflect plan results of the current standards of practice, mainly due to two reasons. First, in this study CTVs instead of PTVs were used as mentioned before. Second, in order to purely investigate the plain impact of the L-based delineation strategy, any high risk nodal and elective volumes were ignored. Therefore, it is difficult to make an assumption in terms of TCP in this simulated scenario. However, the results provide an insight for extra caution possibly needed for target volume delineation for applications like single vocal cord irradiation [10] or stereotactic radiotherapy [11, 12], where one and relatively small target volume is used (either primary tumor or a single lymph node and without elective volumes).

It may be suggested, that the use of image registration techniques with other modalities (e.g. MRI or PET) may overcome the uncertainty whether to use S or L on CT-based volume delineation. However, such an approach causes additional problems. First of all, the image registration algorithms have more geometrical uncertainty than the difference between S and L in CT, on which the radiotherapy plan is generated. In addition to that, these imaging modalities are not free from intrinsic uncertainties caused by problems such as partial volume effect (sometimes varying among different sequences of the same MRI acquisition, for instance caused by different slice thicknesses), varying tumor appearance on MRI due to different acquisition parameters/techniques, movement artefacts (e.g. due to swallowing during MR), different neck positioning compared to planning CT or the lack of agreement over ideal thresholding on PET imaging.

Presumably, the already marginal and (in our subjective opinion) clinically negligible dose differences in OARs between Plan_S and Plan_L would be even less prominent if other high (i.e. involved lymph nodes) and low risk/elective target volumes are to be used. By looking at the overall picture, the results of this study can be interpreted towards two opposite directions. It may be argued that the extra effort to define a GTV_L and CTV_L would be trivial, since CTV to PTV margins of at least 3 mm are used. Today, less than 3-5 mm expansion margins are not recommended to generate PTV from CTV in SCCHN [13, 14], and this may blur and rule out any delineation uncertainties occurring at the air-tissue border, even though these suggested PTV margins do not take the CTV_Δ into account. In a practical sense, an increased PTV expansion based on the HDs arising from CTV_Δ would overcome this problem without increasing the L-based delineation workload. However, this would also unnecessarily enlarge the PTV towards the soft tissue, even by using anisotropic margins. Although the median HD was larger than initially expected, these geometric differences between L and S usually occur in the ‘coves’ of the concave parts of the target volumes neighboring cylindrical air cavities, which are usually engulfed by PTV margins emerging from two tips of the C-shaped volume. It is also worth to note, that using isotropic PTV expansions on CTV_L may increase the dose differences on OARs to a clinically meaningful level and increase toxicity. On the contrary, one may also argue, that the definition of these L-based target volumes does not compromise normal tissue doses in a clinically meaningful way, and poses a more systematic and accurate approach, whereas omitting it may compromise TCP, maybe not in all but some cases.

## Conclusion

Currently, for the routine curative radiotherapy planning of SCCHN, which involves elective volumes and PTV margins, the delineation of L-based volumes seems to be trivial. However, in special settings, where smaller or no PTV margins are used, this approach may play an important role for local control.

## Additional file

Additional file 1: Priorities of the structures for inverse planning optimization and detailed dose results of OARs. (XLSX 46 kb)
